# The effects of community-based education programs on empathy, emotional intelligence, and caring behaviors among nursing students: a scoping review

**DOI:** 10.3389/fmed.2024.1479466

**Published:** 2024-12-12

**Authors:** Ma Jing, Pinglei Chui, Mei Chan Chong, Tang Maoting

**Affiliations:** ^1^Faculty of Medicine, University of Malaya, Kuala Lumpur, Malaysia; ^2^School of Nursing, Henan University of Science and Technology, Luoyang, Henan, China

**Keywords:** nursing students, empathy, emotional intelligence, caring behavior, community-based education

## Abstract

**Introduction:**

Community-based learning approaches are gaining recognition in nursing education as a means to address the needs of aging societies by fostering empathy, emotional intelligence, and caring behaviors among nursing students. These attributes are essential for enhancing the quality of care and building strong interpersonal connections with older adults. While community-based education programs offer promising benefits, there is still limited understanding of their impact on nursing students’ empathy, emotional intelligence, and caring behaviors. This scoping review aims to examine how community-based educational interventions can assist nursing students in developing these essential competencies, ultimately contributing to better outcomes in geriatric care.

**Method:**

A scoping review was conducted following the framework of Arksey and O’Malley. The following electronic databases were searched: Cumulative Index to Nursing and Allied Health Literature (CINAHL), MEDLINE, Web of Science, Psychology and Behavioral Sciences Collection, PubMed, and Google Scholar. Gray literature was also searched through Google Scholar and ProQuest. Studies included reports on nursing students’ emotional changes due to educational engagements with older adults.

**Results:**

A total of 3,001 abstracts were screened, with 78 full texts reviewed, resulting in 9 studies being included in the analysis. The results demonstrate that interactions with older adults enhance nursing students’ empathy and emotional intelligence while fostering positive changes in their caring behaviors. Participants reported greater sensitivity to the feelings and physical discomforts of older adults, contributing to improved care and stronger relational dynamics.

**Discussion:**

Community-based education programs involving older adults represent an effective strategy for enhancing nursing students’ empathy, emotional intelligence, and caring behaviors, suggesting valuable implications for nursing education methodologies.

## Introduction

Community-based education serves as a pathway to address the educational needs of the community and enhance healthcare systems by training healthcare personnel in both developing and industrialized nations, aligning education with community requirements. A community-based educational program consists of an adequate amount of learning activities in a range of well-balanced educational venues. A community learning plan requires that teachers fully consider the needs of community learning and reasonably arrange curriculum content and activities in the process of curriculum planning (1). Teachers should provide students with as many opportunities as possible to participate in community learning activities, and through these activities, students can obtain real and meaningful experiences, make them feel that they are members of the community, and then cultivate their sense of responsibility and mission to society (2, 3). Community-based education programs are increasingly used in professional training for nurses to provide care and social support to various populations, particularly older adults (4). The literature has primarily focused on nursing student groups rather than community care recipients. Community-based education is a valuable method for improving clinical skills, providing technical assistance, and enabling social contact (5). This type of education has the potential to enhance empathy, emotional intelligence, and caring behaviors (6–8).

As the number of studies highlighting the potential advantages of community-based education in geriatric nurse training continues to rise, there is increased interest in exploring its benefits for improving and supporting patient care (9). While existing research suggests that community-based education can be an effective and cost-efficient method for nursing students to enhance their communication skills and social awareness, there remains a significant gap when it comes to identifying programs that support the development of empathy, emotional intelligence, and caring behaviors (5, 10, 11). Despite previous literature reviews exploring the use of social services or community-based education for improving empathy in student nurses, no studies have yet examined the role of community-based education in promoting such skills, specifically when it comes to geriatric care (12). This review thus aims to bridge that gap by offering a comprehensive examination of the evidence surrounding the efficacy of community-based education programs in promoting empathy, emotional intelligence, and caring behaviors in the context of geriatric nurse training.

In the field of nursing education, both empathy and emotional intelligence are essential aspects that encompass various dimensions including moral, cognitive, and behavioral enlightenment (13–17). Amidst the current emphasis on community nursing practice in nursing education focuses more on active learning in real situations to enhance nursing students’ social–emotional awareness of poverty and illness among individuals and families (18), community-based education emerges as a strongly recommended approach in pedagogical innovations. As society rapidly ages, projections indicate that by 2025, one in five Chinese individuals aged 60 and above may experience multiple chronic illnesses, primarily within the confines of their homes (19). This implies that nursing students will have to regularly engage with these elderly individuals within communities.

The current teaching approach is mainly based on classroom teaching, which often does not achieve the nursing students’ learning outcomes on empathy and emotional intelligence (20). Research conducted by Zhao and Kong (21) investigated the empathy level of undergraduate nursing students in Hubei Province, China found nursing students struggle to identify or respond to changes in others’ emotions, as evidenced by their low scores on the “on others’ pain” item. According to another study conducted in China, nursing students in placement had an average emotional intelligence score of 69.29 out of a possible 100 points (22).

Empathy, emotional intelligence, and caring behaviors are recognized as primary elements to improve care outcomes. After investigating 362 undergraduate nursing students, Liu (23) concluded that the empathy ability of undergraduate nursing students and the willingness to serve older adults were both at a medium level, and the two were positively correlated. In other words, improving the empathy ability of undergraduate nursing students can improve their willingness to serve older adults, which is extremely beneficial for China with its rapidly aging population. Emotional intelligence and empathy can help improve moral sensitivity and caring behaviors (24). Nursing students’ empathy is related to personality and emotional intelligence, which can be improved by cultivating their personality and emotional intelligence (25). The higher the level of emotional intelligence of nursing students, the stronger their caring ability (26). It is suggested that educating students about aging and giving them more opportunities to interact with older adults will develop empathy, eliminate negative attitudes, produce a greater willingness to care for old adults, and thereby boost their interest and motivation to provide care and service to them (26). The scoping review method is appropriate for addressing the research question/objectives because it provides a comprehensive overview of the literature on a specific topic (27). The priority is given to including a wide range of studies, not necessarily empirical, which enables a comprehensive understanding of the topic. Furthermore, scoping reviews can aid in identifying important concepts, definitions, and gaps in the available evidence, which can guide future research directions (28). Regarding this review, the objective is to map the evidence on how community-based education programs for older adults can be used to improve empathy, emotional intelligence, and caring behaviors among nursing students.

## Methods

### Study design

This scoping review was based on the approach described by Arksey and O’Malley (29) and further developed by Levac, Colquhoun (30). This systematic review method is recommended by the Joanna Briggs Institute as it improves the review’s rigor and clarity (31). This procedure entails a 5-step process that includes the following steps: (1) establishing and aligning the objective(s) and questions(s); (2) developing and matching the inclusion criteria with the objective(s) and question(s); (3) outlining the planned approach from evidence searching, selecting data extracting, to presenting the evidence; (4) summarizing the evidence as to the review’s purpose, (5) drawing a conclusion, and noting any implications that the results have. This scoping review was registered on Open Science Framework, with registration DOI number https://doi.org/10.17605/OSF.IO/JK7EB. The PRISMA reporting guidelines was followed to provide guidance for the reporting of this scoping review by evaluating the effects of interventions (32). This scoping review was structured and organized according to the PRISMA checklist, ensuring that all relevant components were systematically addressed. Following these established guidelines enhances the clarity and completeness of the review, providing a robust foundation for the findings and conclusions. Adhering to PRISMA reflects a commitment to maintaining high standards in research methodology.

### Identifying research question

The question “What is the existing literature on the use of community-based education programs for older adults to support empathy, emotional intelligence, and caring behaviors development among nursing students?” was acted as a starting point to provide search terms. According to the PICO framework for formulating clinical questions, the queries include four aspects: Patient-Problem (P), Intervention (I), Comparison (C) and Outcome (O). In this regard, Population, Intervention, Comparators, Outcomes, Study Design (PICOS) criteria were used for this study: population (undergraduate nursing students and medical students), intervention (community-based education and geriatric curriculum innovation) and outcome (empathy, emotional intelligence, caring behavior).

### Inclusion and exclusion criteria

Following the PCC framework (Population, Concept, and Context) (33), studies were included if they were: (1) English language articles that were published in the past 10 years, from 2013 to 2024; (2) primary research articles about community-based education, and examined the changes in the undergraduate nursing students’ empathy, emotional intelligence, and caring behaviors (dissertations, peer-reviewed research, evidence-based practice guidelines); (3) primary research that looking into the effectiveness of the older adults’ education for improving nursing students’ capabilities or competencies; and (4) articles related to community-based medical education. The search also included gray literature, such as conference reports, graduate thesis, and so on.

The exclusion criteria were: (1) studies that did not answer the main research question or meet the main objective of this scoping review (for instance, the studies that focused on the effects on older people rather than on nursing students); (2) an inappropriate study design (for example, observational studies); (3) studies that were not published in peer-reviewed journals or in conference reports; (4) studies not published in English; and (5) outdated or insufficient information included in the document.

### Search strategy: approach, evidence searching, selected data extracting

The literature search was performed using English databases including the Cumulative Index to Nursing and Allied Health Literature (CINAHL), Web of Science, MEDLINE, Psychology and Behavioral Sciences Collection, PubMed, and Google Scholar. These databases were selected for their information contained on nursing education.

A three-step search strategy was implemented (33). Initially, to make sure no synonyms had been missed in the search strategy, the author and another researcher who have studied systematic reviews of literature search methods and methods of evaluating the quality of literature double-checked the Medical Subject Headings (MeSH) in PubMed and keywords that showed in the results from the initial search. The keywords used for the search were “medical student* “[MeSH terms] OR “nursing student* “[MeSH terms] OR “students, health occupations” [MeSH terms]; “community-based” [Title/Abstract] OR “service learning” [Title/Abstract] OR “service education” [Title/Abstract] OR “community education” [Title/Abstract]; “Education” [MeSH terms]; “Geriatric Nursing” [MeSH terms] OR “Senior Centers” [MeSH terms] OR “Geriatric care” [MeSH terms]; OR “Elderly” [MeSH terms] OR “Older adult*” [Title/Abstract] “Gerontologic” [MeSH terms] “Curriculum” [MeSH terms] OR “Problem-Based Learning” [MeSH terms]; “Empathy” [MeSH terms]; “Emotional Intelligence” [MeSH terms]; “caring behavior*” [Title/Abstract]. The keywords were searched as individual words and in combination using the Boolean phrase such as “AND” and “OR.” Boolean operators such as “AND,” “NOT” and “OR” were employed to combine or exclude keywords. These helped to connect the keywords during the search, to either narrow or broaden the results. In addition, secondary citations from books and journals were also used.

Secondly, more keyword synonyms were included in the search phrases in the listed databases. Thirdly, eligible publications were found by manual-searching reference lists from the included research. This was followed by a Google search of gray literature. Two distinct researchers conducted the searches and then compared and evaluated the records for inclusion. Loading citations into the EndNote 20.0 reference manager program simplifies the process of removing duplicates and screening articles for complete text. The remaining studies were examined for relevance and compared to the inclusion criteria.

## Results

### Results of search with procedures of diagram

**Figure 1:**
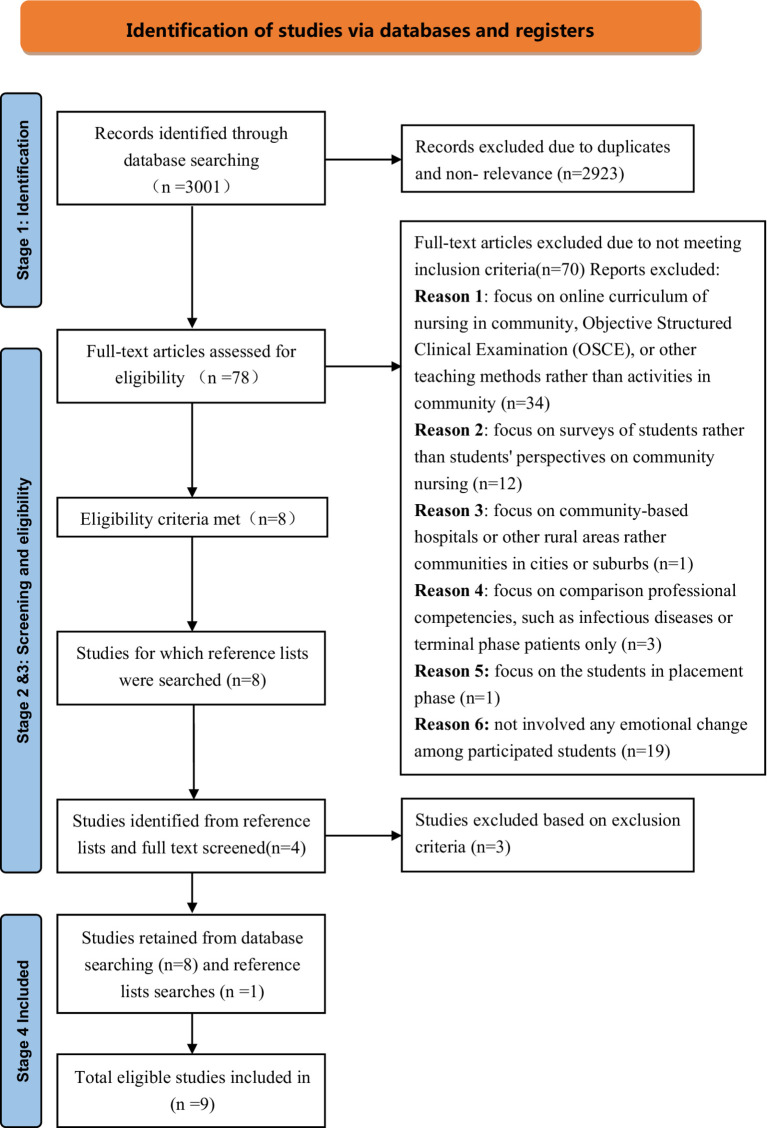
PRISMA flowchart elaborating identification, screening, and eligibility.

In accordance with the Preferred Reporting Items for Systematic Reviews and Meta-Analysis extension for Scoping Reviews (PRISMA-ScR), the researcher completed the search strategy, evidence screening and selection, data extraction, and data analysis (34). Figure 1 illustrates a PRISMA flowchart that visually outlines the processes of study identification, screening, and eligibility assessment related to the specified research question. Although the review primarily focuses on literature from the past ten years (2013–2024), older seminal studies have been included where necessary to provide a comprehensive understanding of the foundational concepts and to trace the historical development of community-based education for nursing students. This approach aligns with the flexibility inherent in scoping review methodologies, as highlighted by Munn (35), which prioritize inclusivity and a broad range of evidence to address the research objectives. A total of 3,001 records were identified and retrieved from 6 electronic databases: CINAL (*n* = 28); PubMed (*n* = 86); Web of Science (*n* = 2,175); MEDLINE (*n* = 211); Psychology and Behavioral Sciences Collection (*n* = 412); and Google Scholar (*n* = 89).

During the initial screening, 390 duplicated articles and 787 irrelevant articles were deleted, while 725 items were not retrieved. After being evaluated for eligibility, 1,021 of these records were excluded because they did not meet the inclusion criteria, specifically, that they did not investigate or assess a communication-based older people education method to support nursing students’ empathy, emotional intelligence, or caring behaviors and were mostly discussion articles. A total of 2,923 articles were excluded during this process. The full text of the remaining 78 remaining papers was then evaluated, and eight of them met the inclusion criteria. The reference lists of these eight articles were further examined for relevant studies, resulting in the identification of four additional studies. However, three of them were excluded based to the criteria. One more article was included in the reference lists. Finally, the narrative synthesis includes 9 suitable full-text articles all published between 2013 and 2024. The quality appraisal scores for the included studies ranged from 70 to 100%, based on the guidelines developed by Caldwell, Henshaw (36).

### Summarizing the evidence

Publication dates were from 2013 until 2024, with research originating from the United States (*n* = 3), Canada (*n* = 1), Malaysia (*n* = 1), England (*n* = 1), Spain (*n* = 1), and Taiwan (*n* = 2). The total number of nursing students who participated in these nine studies was 651. Six studies were qualitative (67%), and three mixed method studies (33%).

### Selection of studies

The study selection involved a two-step process for data extraction. Two researchers independently screened the titles and abstracts based on predetermined criteria. Afterward, the remaining articles were subjected to full-text screening. For each eligible study, two reviewers independently extracted key information using a standard data extraction form, including the first author’s name, country, year of publication, study design, study aims and objectives, study population, intervention, the key findings, and limitations. Any discrepancies were resolved by a third reviewer. To record study characteristics, a data extraction table was created, quality score and key findings relevant to the review question are included in Table 1.

**Table 1:** Characteristics of the studies included in the scoping review (*N* = 9).

The first author, year, country	Study aims and objectives	Research design	Quality score	Study population and intervention	Key findings relevant to the review question	Limitations
Hwang, 2014 (43), Taiwan.	To describe the development of a service-learning project that spans generations and to evaluate its effects on nursing students who are partnered with residents and residents of facilities.	Mixed method design	88	Population: 210 nursing students Intervention: 120 h of intergenerational service learning (ISL).	ISL interventions are essential in long-term care education programs. ISL promotes considerate behaviors toward the elderly. After the intervention, students displayed increased caring behaviors and more positive attitudes toward the elderly in a post-test. The students found the experience of understanding and interacting with elderly citizens’ feelings to be the most rewarding. Through interaction with residents, students met the requirements of the program while enhancing their learning.	The universality of the data in other projects and long-term care facilities need to be further verified.
Brown K. M. and Bright L. M., 2017 (40), United States.	To investigate the experiences and attitudes of students toward older adults who have cognitive and/or physical disabilities, and find the effects on students’ knowledge and skills during a baccalaureate nursing course which included service-learning experience.	Post-test cohort study	83	Population: 45 students, 24 junior-level baccalaureate nursing students, and 21 senior-level students. Intervention: a specific SL nursing course focus on older adults	Data saturation was reached by the end of the Service Learning (SL) course with older adults. Nearly every participant expressed positive feelings about the interactions. Perceptions of the SL experience included being pleasantly surprised by positive feelings toward older adults. Emotions associated with the experience included happiness, caring, compassion, respect, empathy, and appreciation for older adults.	A convenience sampling method was used, and not everyone participated in the member check.
Pesut et al., 2015 (47), Canada.	To investigate if a collaborative educational intervention could enhance the capabilities of nursing students in supportive care by enhancing their care of clients in the community.	A mixed-method study	83	Population: 21 nursing students and 15 community clients. Intervention: two-day workshop and a 12 week innovative clinical experience	Registered Nurse (RN) students experienced more positive changes in spiritual needs, ethical and legal issues, and care during the last hours of life. Health Care Assistant (HCA) students showed greater improvements in managing physical symptoms. Mean self-perceived confidence levels increased for all participants after the workshop. However, interprofessional collaboration, communication, and personal and professional relationships fluctuated during the clinical experience.	The program was implemented in a rural context, where pre-existing relationships might have influenced the outcomes. Despite efforts to provide creative placements to enhance nursing duties, students may not have been adequately prepared in standard competencies due to the absence of a strong nursing presence.
Hsu P. T., 2022 (37), Taiwan.	To investigate how the integration of service learning and portfolio construction into the gerontological nursing curriculum affects nursing students’ attitudes toward aging and older people’s behavioral intentions.	Mixed method experimental study	94	Population: 82 students across all stages. The experimental group consisted of 41 students. Similarly, the control group also comprised 41 students. Intervention: four visits to community elder care stations combined with 10 weeks of learning integrated with the gerontology nursing curriculum for experimental group, while the control group received traditional lecture-based aging courses.	A student expressed learning to communicate with older people and developing compassion through service learning. They expressed enjoyment in getting to know the elderly and a future intention to work in the care of senior citizens. Students reported that creating portfolio content and reflecting on their learning improved their views on aging, increased empathy, and prepared them to assist the elderly in the future.	The study’s findings on changed attitudes toward aging and older people’s behavioral intentions are limited. The study lacked a follow-up period. More research is needed to determine the consistency and applicability of the findings to other student demographics.
Alex et al., 2021 (42), Malaysia.	To incorporate elderly care topics into the current curriculum for undergraduate medical students, an introductory novice-level geriatric module was created.	Qualitative study	77	Population: 143 medical students Intervention: three weeks module with collaborative active learning sessions on healthy aging, interaction with older adults from the community, and a simulated experiential activity	The care of the aged module had a positive immediate effect on medical students. Reflections from students indicated they were better able to relate to and empathize with the elderly after the sessions. They also expressed understanding the frustration and helplessness experienced by the elderly on a daily basis. Students recognized the importance of verbal and non-verbal cues when communicating with elderly patients.	The approach is not innovative enough and cannot meet the challenges of an aging population.
Walton and Blossom, 2013 (39), United States.	To explore the experiences of nursing students working with older adults in rural communities, the experiences of older adults, and the development of relationships.	Qualitative study.	88	Population: 96 nursing students and 16 older adults in the community. Intervention: 7 and more times of home visiting in one semester.	Initially, students experienced anxiety before interacting with older adults. However, by the end of the experience, they felt confident and gained insight into aging. They were able to establish therapeutic relationships and felt welcomed and valued. Older adults felt honored and valued as mentors to student nurses, allowing them into their homes to build relationships and practice skills. This partnership enriches the lives of older adults in rural communities and the student nurses who visit them.	Many students felt anxious about completing a home safety assessment during their first home visit. Guidelines suggested that students should not be suddenly exposed to older people’s homes. It is difficult to compare with absent students as a qualitative study. There were no precise data on perceptions toward aging.
Dobarrio-Sanz et al., 2023 (41), Spain	To explore the experience of nursing students participating in a home visiting program for community-dwelling older adults with chronic multimorbidity.	Qualitative study	22	Population: 22 nursing students. Intervention: Forty hours of specific training and 12 h of face-to-face clinical simulation sessions before home visiting. Then visit the old people once a week lasting 30 to 45 min for 12 weeks.	Nursing students were skeptical and had been unenthusiastic before the start. They built a trusting relationship with the old people and felt motivated and rewarded as they were providing real holistic care. The students felt a moral and professional obligation to learn after they viewed the elderly’s health declining. The direct practical experience reinforced the theoretical learning among nursing students, which enabled them to pass the module test more easily. They acquired competence and communication skills during the situations. And developed in-depth attitudes, such as respect, empathy, and active listening toward older adults. Personal perspectives had been changed and the nursing students behaved like qualified nurses since the real disabled people’s willingness was being explored by themselves.	The study’s limitations include a homogeneous sample of white Spaniards and data collection limited to post-program interviews, suggesting that a more diverse sample and multiple interviews throughout the program could have provided broader insights.
Shropshire et al., 2022 (38) United States	To explore the perceptions and emotions of undergraduate nursing students toward older adults living in nursing homes prior to their initial clinical encounter with these residents.	Qualitative research	72	Population:20 participants Intervention: 7 weeks of clinical component when learning the Nursing Fundamentals course.	Participants shared some moments of nervousness, excitement, frustration, and confidence with nursing skills feelings, and felt that future work would have more challenges. The students described beautiful, reciprocal relationships with older adults. They also provided compassionate care while learning a lot from the elderly. The practical session in the nursing homes also helped the students with confidence enhancement, competency skills development, and career preparations. Twelve of the 20 participants expressed the desire to supply the best care for the elderly in the future work and change the negative attitudes of the older adults. The experience with older people aroused nursing students’ concerns about the environment of nursing homes and the rights of the elderly, and they communicated how to care for older people better.	The study’s limitations included the analysis of course artifacts from only two iterations of a single course at one university, lack of demographic data or detailed information beyond the original assignment prompt, limited transferability due to the narrowness of data, variability in students’ response depth, and potential bias in responses aimed at pleasing faculty despite the assignment not being graded.
Grosvenor et al., 2021 (44), England	To explore the effect of the Time for Dementia Program that puts dementia patients and their family carers in the position of mentors and educators for nursing students.	Qualitative research	Twelver undergraduate students	Population: 12 undergraduate nursing students. Intervention: 60 min of interviews of visiting people with dementia and 28 interviews were carried out at three yearly points.	The nursing students developed a Whole Sight by stepping into the shoes of people with dementia and their caregivers, which resulted in their reframing perceptions of dementia. Greater self-awareness of the importance of holistic care was developed, and the students began to know how to listen to one’s talk. Adaptive thinking, relationship building with patients, and transformative learning were all interrelated by the results revealed. Participants were capable of being with a person and engaging with the person instead of just doing which helped them work as a nurse.	A limitation of this study is that the sample was limited to adult nursing students in one higher education institution, resulting in a small number of participants, limiting the potential for generalization of the findings to the wider health care training population.

### Quality appraisal and evidence presentation

The critical assessment methodology proposed by Caldwell, Henshaw (36) was followed to conduct the assessment of the included literature. To analyze the advantages and disadvantages of research using qualitative, quantitative, and mixed-method designs, each paper was evaluated against 18 appraisal criteria. To determine how well each paper satisfied the quality appraisal guidelines, a percentage score was computed. The outcomes can be referred to in the quality assessment table shown in Table 2. Assessments of the risk of bias due to missing results were conducted, and each synthesis was assessed. A narrative review was employed to synthesize knowledge. Through this approach, relationships in data could be discursively explored and findings could be compared across studies using divergent methodologies.

**Table 2:** Checklist for quality assessment.

Qualitative studies	1. Hwang (43)	2. Brown K. M. and Bright L. M. (40)	3. Pesut et al. (47)	4. Hsu P. T. (37)	5. Alex et al. (42)	6. Walton and Blossom (39)	7. Dobarrio-Sanz et al. (41)	8. Shropshire et al. (38)	9. Grosvenor et al. (44)
1. Does the title reflect the content?	√	√	√	√	√	√	√	√	√
2. Are the authors credible?	√	√	√	√	√	√	√	√	√
3. Does the abstract summarize the key components?	√	√	√	√	√	√	√	√	√
4. Is the rationale for undertaking the research clearly outlined?	√	√	√	√	√	√	√	√	√
5. Is the literature review comprehensive and up to date?	×	×	×	√	√	×	√	×	×
6. Is the aim of the research clearly stated?	√	√	√	√	√	√	√	√	√
7. Are all ethical issues identified and clearly addressed?	√	√	√	√	√	√	√	√	√
8. Is the methodology identified and justified?	√	√	√	√	×	√	√	√	√
9. Are the philosophical background and study design identified and the rationale for choice of design evident?	√	√	√	√	×	√	√	√	×
10. Are the major concepts identified?	√	√	√	√	√	√	√	√	√
11. Is the context of the study outlined?	√	√	√	√	√	√	√	√	√
12. Is the selection of participants described and is the sampling method identified?	√	×	×	×	×	√	√	√	×
13. Is the method of data collection auditable?	√	√	√	√	√	√	√	√	√
14. Is the method of data analysis credible and confirmable?	√	√	√	√	√	√	√	√	√
15. Are the results presented in a way that is appropriate and clear?	√	√	√	√	√	√	√	√	√
16. Is the discussion comprehensive?	√	√	√	√	√	√	√	√	×
17. Are the results transferable?	√	√	√	√	√	√	√	√	√
18. Is the conclusion comprehensive?	×	×	×	×	×	×	√	×	×
Total quality score as a percentage:	88	83	83	88	77	88	100	88	70

Quantitative studies	1. Hwang (43)	2. Brown K. M. and Bright L. M. (40)	3. Pesut et al. (47)	4. Hsu P. T. (37)	5. Alex et al. (42)	6. Walton and Blossom (39)	7. Dobarrio-Sanz et al. (41)	8. Shropshire et al. (38)	9. Grosvenor et al. (44)
1. Does the title reflect the content?	√		√	√					
2. Are the authors credible?	√		√	√					
3. Does the abstract summarize the key components?	√		√	√					
4. Is the rationale for undertaking the research clearly outlined?	√		√	√					
5. Is the literature review comprehensive and up-to-date?	×		×	√					
6. Is the aim of the research dearly stated?	√		√	√					
7. Are all ethical issues identified and clearly addressed?	√		√	√					
8. Is the methodology identified and justified?	√		√	√					
9. Is the study design clearly identified, and is the rationale for choice of design evident?	√		√	√					
10. Is there an experimental hypothesis clearly stated? Are the key variables clearly defined?	√		×	√					
11. Is the population identified?	√		√	√					
12. Is the sample adequately described and reflective of the population?	√		×	√					
13. Is the method of data collection valid and reliable?	√		√	√					
14. Is the method of data analysis valid and reliable?	√		√	√					
15. Are the results presented in a way that is appropriate and clear?	√		√	√					
16. Is the discussion comprehensive?	√		√	√					
17. Are the results generalizable?	√		×	√					
18. Is the conclusion comprehensive?	×		×	×					
Total quality score as a percentage:	88		77	94					

## Discussion

The effects of a community-based education program for older adults on nursing students are discussed under five subheadings: empathy improvement was observed in the literature; emotional intelligence improved during the interaction; caring behavior changes were observed after the interactions with the older adults; nursing students showed sensitivity to others’ feelings and physical discomfort, which triggered better care; a solid and relaxing relationship developed through inter-generational interactions. The results presented include both community-based education programs and service-learning programs since the themes identified across all papers did not significantly differ.

### Empathy improvement was observed in the literature

Nursing students showed empathy improvement through the curriculum intervention aimed to improve the students’ “aging-related awareness,” “feeling toward older adults” and “interpersonal interactions with older adults” (37, 38). Negative emotions were observed to diminish during the interactions; students stated that their perspective had completely changed with regard to older adults (38). The dissolution of age-related stereotypes was one of the developments. Before the educational intervention, most of the nursing students held the opinion that “older adults are often frail and sick, they often have a patronizing attitude and are difficult to get along with, they are frequently disconnected from normal day-to-day life, they have poor work efficiency.” These original attitudes were also noticed in the study by Walton and Blossom (39). However, as the nursing students began to realize that the older adults were easy to get along with and well-mannered, their first impressions changed. Their attitude became more positive as they better understood and began to respect the selflessness of the older adults (40). The students reported a deeper emotional connection and a better appreciation of the challenges faced by older adults (41). Meanwhile, they realized that the older adults who were in their 80s were, nevertheless, still capable of caring for themselves, with some medical support. The interaction with older adults allowed students to learn directly from their experiences, which fostered a more compassionate and empathetic approach to care (38). Ultimately, the nursing students expressed astonishment at and appreciation for their aged charges, in contrast to their previous stereotypes (39). Other positive feelings that came about from these interactions with older people included compassion, caring, happiness, respect, empathy, appreciation of older adults, insightfulness, inspiration, gratitude for the encounter, and appreciation for the life-changing experience (42, 43).

### Emotional intelligence improved during the interaction

Students recognized the value of what they were learning from older adults. They gained a closer understanding of the community resources and a closer look at the socioeconomic determinants of health. Students’ admiration for people with disabilities was sparked by their efforts to overcome limitations, such as the woman who cooked and baked with just one arm (41). During the participation in the “Time for Dementia” program, nursing students learned to better navigate their emotional responses and understand the emotions of dementia patients (44). Similar changes were also reported by Shropshire et al. (38) and Dobarrio-Sanz et al. (41), participants in the research noted a marked increase in their ability to empathize and understand the emotional states of older adults. Nursing students also gained valuable first-hand knowledge about the social determinants of health and living with chronic disease. They learned how to define the occasions to listen rather than interrupt or pose solutions for clients. When dealing with someone who is aging and growing less and less capable in his or her place, visiting older adults at home differs from institutional care in that it involves a sense of giving and being personally involved (40). In terms of older adults’ frustrating changes due to aging such as physical disability, students understood the significance of older adults advocacy using non-verbal communication and silent observation (37). During their weekly interactions, the nursing students eventually learned to trust their charges and converse empathetically with them (43). Most of the students and older adults became friends, with a sense of mutual understanding arising between them (37). Finally, the nursing students began to approach the topics of age and change with a constructive perspective, and there was a sense of achievement and a feeling of cheerfulness. They obtained the pathway to communicating with older adults with high emotional intelligence (37). Nursing students were able to address older adults’ concerns in a better way and so would likely appreciate any opportunity to encounter an older adult in the future.

### Caring behavior changes were observed after the interactions with older people

The caring behaviors of the nursing students improved over the course of communicating with the older people. The mean score of the caring behaviors improved from 50.25 ± 9.30 to 55.75 ± 8.62, while the attitude improved from 101.9 ± 10.85 to 106.12 ± 11.52 (43). Nursing students realized the importance of having better caring skills with older adults as they truly believed that they needed to equip themselves well for work with the elderly, which would be more likely in the future (42). They learned how to create a comfortable therapeutic relationship while also improving their listening and communication skills. Not only talking but also feeling comfortable with silence, which also showed their willingness simply to “be with.” The nursing students observed the value of silence in therapeutic communication. Even limited encouragement and attentiveness were quite helpful (39). Moreover, the experience led to personal and professional growth, emphasizing person-centered care through practice, and students reflected that they were more confident in learning nursing knowledge, which will induce high-quality care (41). Caring holistically is essential to the maintenance of a therapeutic relationship. The nursing students often discussed life stories with the older adults and learned the value of hard work from them, which also inspired them to enhance their caring behaviors by exploring the skills to relax and comfort the older adults during their connection.

### Nursing students showed sensitivity to others’ feelings and physical discomfort

Students who participated in the supportive care project for older adults living with advanced chronic illness became increasingly aware of their psychological and spiritual needs, were more sensitive to ethical and legal issues, and more competent in communication. Even though they also showed concerns about old adults’ pain and other physical symptoms, they showed more subjective emotions or feelings (37, 40). By involving two senior citizens from the community in the active learning session, the nursing students realized that older people have different needs compared to the general population. They also understood how individual experience plays an integral role in managing the patient as a whole. They adapted their daily life to show their respect and did not just do everything for them. They became more confident as they better empathized and related to their elderly charges after each active session (42). The nursing students’ attitude to older adults complaining about their constant aches and pains changed from assuming they were exaggerating the discomfort to now trusting their word and empathizing with their frustration and feeling of helplessness (42).

### A solid and relaxing relationship developed through inter-generational interactions

Nursing students went into the experience of the course with a lack of confidence and nervousness but came out of it with confidence and self-assurance. The older participants were referred to by the nursing students as their “health partners.” They always welcomed students and offered hospitality. They invariably greeted each other with hugs and kisses, which brought fun and mutual respect to the interaction (38). Through these enjoyable visits, a trusting bond between the students and the older adults quickly formed. A turning point was sometimes reached when a talk of a spiritual nature occurred, or some other meaningful social interaction happened, which meant a deeper understanding then developed between the two (40). Some students said they even felt a type of love grow between them, and it was a sad day when the program, and their contact, finally ended (39). This experience provided nursing students with more knowledge and background for detecting nonverbal cues. After a while, they could tell by the older person’s expression and voice whether they were happy or sad. They understood what it meant to listen and were more sensitive to the unique needs of each patient (39).

## Strengths, limitations, and recommendations

This scoping review offers a comprehensive mapping of the evidence on community-based education programs for nursing students, with a particular focus on their impact on empathy, emotional intelligence, and caring behaviors in geriatric care. A key strength of this review lies in its inclusive approach, incorporating a wide range of study designs and methodologies to ensure a broad understanding of the topic. By synthesizing evidence across diverse cultural and contextual, the findings provide valuable insights into the applicability of community-based education in different settings. Additionally, the review examines how cultural background differences can influence emotional reactions during interactions, highlighting their impact on student development. These nine studies were carried out in North America, Europe (Spain, England), Southeast Asia, and East Asia, representing different cultural differences, that is, from Western and Eastern perspectives. The diversity of different regions and cultures makes this scoping review more informative. Additionally, the cultural differences between the East and the West shape nursing undergraduates’ diverse attitudes toward the elderly. This has the potential to bias the research findings. Because compared with the unadulterated attitude toward the elderly without affection from other factors in the West, the respect for older adults, kindness and feedback cultural education from a young child in the Eastern countries may bias the research results in East Asia, as the cultural influence has been deeply rooted in nursing students, which may cover up the true level of empathy, emotional intelligence, and caring behaviors.

However, limitations should also be acknowledged. First, the review primarily includes literature published in English, which may have excluded relevant studies published in other languages. Second, despite a rigorous search strategy, the lack of empirical studies specifically focused on geriatric-focused community-based education limits the ability to draw definitive conclusions. Thirdly, the potential for publication bias, as only published literature was reviewed, may have excluded unpublished but relevant studies.

To better equip future nursing professionals with essential humanistic qualities, it is crucial to promote further research on effective methods for fostering empathy, emotional intelligence, and caring behavior within educational settings. Currently, nursing educators tend to prioritize the improvement of nursing procedures and the dissemination of health information. Although they acknowledge the importance of training in humanistic qualities, few have received systematic education on the methods to enhance emotional perception and sensitivity. As a result, many lack the skills necessary to explore and effectively address the development of empathy and emotional intelligence in nursing education (45). Only a limited number of papers related to empathy and emotional intelligence training, including actual teaching activities, have been published. Although caring is regarded as the basis of nursing, the education of caring behavior can only be embodied in certain scenarios and cannot be separated from situations, so at present, there is a lack of analysis and understanding of it and a dearth of related papers (46). One thing to keep in mind while planning and analyzing research on the consequences of helping older adults in the community is that if research participants are aware that the research team is watching them, the Hawthorne effects could happen. The impact of this effect on these studies is unknown, but it appears important to emphasize the research team’s independence from assessing course completion and the confidentiality of participants. A related issue in intervention design is whether and how to include nursing teachers, as this may directly influence nursing students’ attitudes and behaviors, limiting the openness with which they communicate and express their emotions toward older people. It is expected that more nursing educators will pay attention to the research of empathy, emotional intelligence, and caring behavior, to offer guidance for training students on how to provide more humanized nursing.

## Conclusion

This scoping review is the first to map out how community-based programs for older adults can be used to support empathy, emotional intelligence, and caring behaviors among nursing students. The findings suggest that community-based education programs for older adults may have the potential to improve nursing students’ empathy, emotional intelligence, and caring behaviors. It can provide an accessible and cost-effective means of supporting emotional intelligence development and nurturing, caring behaviors among nursing students on campus whose study is usually restricted in a purely academic environment. Such innovations could be beneficial to nursing students’ communication skills, fostering responsibility for social service, and providing better care for older adults. Further research is needed in this area, as this review highlights research involving the development of humanistic care competencies in nursing students. The individual study interests, social requirements, and educational backgrounds of nursing students in different geographic areas should be considered when conducting future research in this area. Standards of care in aging societies can be improved by further research into how community-based education programs for older adults can be used to support the development of specialist geriatric nurses. Community-based education programs for older adults and how they benefit nursing students may have implications for the curriculum of nursing professionals around the world.

## Data Availability

The raw data supporting the conclusions of this article will be made available by the authors, without undue reservation.
